# Prenatal stress effects in a wild, long-lived primate: predictive adaptive responses in an unpredictable environment

**DOI:** 10.1098/rspb.2016.1304

**Published:** 2016-09-28

**Authors:** Andreas Berghänel, Michael Heistermann, Oliver Schülke, Julia Ostner

**Affiliations:** 1 Department of Behavioural Ecology, University of Göttingen, 37077 Göttingen, Germany; 2 Endocrinology Laboratory, German Primate Center, 37077 Göttingen, Germany; 3 Research Group Primate Social Evolution, German Primate Center, 37077 Göttingen, Germany

**Keywords:** resource allocation, gestational stress, developmental constraints, silver spoon, life history, developmental origins of health and disease

## Abstract

Prenatal maternal stress affects offspring phenotype in numerous species including humans, but it is debated whether these effects are evolutionarily adaptive. Relating stress to adverse conditions, current explanations invoke either short-term developmental constraints on offspring phenotype resulting in decelerated growth to avoid starvation, or long-term predictive adaptive responses (PARs) resulting in accelerated growth and reproduction in response to reduced life expectancies. Two PAR subtypes were proposed, acting either on predicted internal somatic states or predicted external environmental conditions, but because both affect phenotypes similarly, they are largely indistinguishable. Only external (not internal) PARs rely on high environmental stability particularly in long-lived species. We report on a crucial test case in a wild long-lived mammal, the Assamese macaque (*Macaca assamensis*), which evolved and lives in an unpredictable environment where external PARs are probably not advantageous. We quantified food availability, growth, motor skills, maternal caretaking style and maternal physiological stress from faecal glucocorticoid measures. Prenatal maternal stress was negatively correlated to prenatal food availability and led to accelerated offspring growth accompanied by decelerated motor skill acquisition and reduced immune function. These results support the ‘internal PAR’ theory, which stresses the role of stable adverse internal somatic states rather than stable external environments.

## 1. Introduction

The role of prenatal maternal stress in the concept of the developmental origins of health and disease receives a great deal of attention [1–5]. Numerous animal and human studies have shown elevated prenatal maternal physiological stress (i.e. elevated prenatal maternal glucocorticoid levels; PreGC) in response to a wide range of external adversities like predation, climatic, social or nutritional stress, and PreGC to cause various effects on offspring phenotype [1–8]. It remains unclear whether prenatal maternal stress effects on offspring phenotype are adaptive from an evolutionary point of view [1,5,8–11]. Current adaptive theories propose that under adverse conditions, these effects benefit the offspring either in the short (developmental constraints) or in the long term (predictive adaptive responses, PARs). The developmental constraints hypothesis predicts the offspring to reduce its investment into development, and particularly growth, to reduce starvation risk under adverse conditions. The PAR hypothesis proposes that PreGC prepares the offspring for its likely long-term future. From life-history theory, the PAR hypothesis predicts that under adverse conditions, the offspring should accelerate its growth, maturation, reproduction and general pace of life, because adverse conditions are likely to reduce offspring's life expectancy [1,11–17]. Under limited resources, such an adaptive recalibration may be part of a life-history trade-off and at the expense of more quality-related attributes (like skill acquisition or immune function) the benefits of which accumulate with increasing lifespan [11,17].

The original and most prominent version of the PAR hypothesis proposes that if PreGC changes with prenatal environmental conditions and if prenatal environmental conditions forecast future environmental conditions, then PreGC may adaptively recalibrate the offspring's phenotype to match similar environmental conditions during its adulthood, thereby increasing its fitness compared with unaltered, mismatched phenotypes (external PAR [1]). It has been argued, however, that external PARs rely on rather unrealistically high environmental stabilities particularly in the case of long time gaps between birth and adulthood, and are otherwise not advantageous [12,13,18,19]. In a more stochastic environment, altered offspring phenotypes will face stronger and more frequent adult phenotype–environment mismatches than unaltered phenotypes which match the evolutionary average environment [12,13]. Recently, another subtype of PAR was proposed which leads to similar effects but is independent of environmental (in)stability and only relies on the rather inevitable long-term effect of early life developmental constraints on adult mortality [12,13]. This internal PAR hypothesis proposes that offspring facing PreGC-related developmental constraints could utilize its currently impaired somatic state to predict its rather unavoidably impaired future somatic state and reduced life expectancy, and recalibrate its life-history pace to optimally cope with these constraints in the long term [12,13,16].

Previous studies on various taxa found that PreGC is related to decreasing prenatal food intake and reduced pre- and postnatal maternal physical condition and investment, and thus resource availability for the offspring [8,20–23]. Both PreGC and reduced resource availability were shown to constrain offspring development in terms of reduced offspring growth [24–26], immune function [6,7,21,27,28], skill acquisition [4,6,26,29] and cognitive development or neurodevelopment [2,4,6,9]. These developmental constraints have long-term consequences because adverse early life conditions lead to disadvantaged adult phenotypes including health deterioration, increased mortality, shortened lifespans and reduced reproductive success in long-lived species including humans, roe deer, elephants and baboons [16,17,30–35]. Such detrimental short- and long-term effects question the existence of PARs and suggest that PreGC and early adversity merely lead to developmental constraints and silver spoon effects [30,35]. However, PreGC and early adversity can also lead to increased offspring growth rates or accelerated reproduction even in the same species [11,14,15,25,33,34,36–39], which was claimed to support the existence of PARs [1,11,15].

The ambiguity in these results makes it currently difficult to assess the adaptive value of prenatal maternal effects on offspring phenotype and to differentiate between developmental constraints and PARs [8]. It is also difficult to further distinguish between the internal and external PAR hypotheses, because they differ primarily in whether the mechanism requires high environmental predictability, but make otherwise similar predictions and are mutually compatible [12]. Most studies were conducted on captive animals and humans exposed to highly artificial and/or extreme stressors [24,25,29,35,37], and to environmental predictabilities that may strongly differ from the natural, evolutionarily relevant conditions. Studies on wild animals facing natural ecological conditions and evolutionarily relevant stressors are extremely rare [8,14,28,40,41]. In particular, the occurrence of prenatal maternal stress effects in long-lived species has not been investigated under natural conditions yet, although it represents a critical test case for the internal PAR hypothesis [8,12,13,33,41,42].

This study provides such a test case, presenting the first data on the causes and consequences of a wider range of pre- and postnatal maternal effects in a wild long-lived mammal, the Assamese macaque (*Macaca assamensis*), under natural ecological conditions. We have previously shown for our study group that immatures experience remarkable postnatal developmental constraints, with decreasing postnatal food availability being associated with reduced rates of growth and play, and, consequently, also decelerated motor skill acquisition [26]. Here, we investigate whether prenatal maternal food availability and PreGC lead to mere developmental constraints or a PAR in our study group. Environmental conditions are exceptionally unpredictable in Southeast Asian forests [43–45]. In our study area, year-to-year predictability of food abundance (correlation between successive years *r* = 0.05, *p* = 0.92, over 8 years) and rainfall (*r* = −0.09, *p* = 0.86, over 8 years) are very low. Hence, our long-lived study species lives and probably evolved in a highly unpredictable environment [45–47] where an external PAR would by definition be unlikely to be advantageous, and can therefore be excluded, making it a test case of the internal PAR hypothesis.

We combined observations of offspring behaviour with measures of maternal pre- and postnatal physiological stress (via faecal glucocorticoid measures), quantitative measures of natural food availability, and individual offspring growth rates measured via photogrammetry. To assess whether accelerated growth is accompanied by detrimental effects on more quality-related offspring attributes, we additionally measured offspring motor skill acquisition and used an outbreak of conjunctivitis to non-invasively and roughly assess immune function.

We first predict that prenatal maternal food availability is negatively correlated to PreGC, which might be further associated with maternal rank and offspring sex [1–3,10,36]. In the case of a PAR, we predict that PreGC leads to increased postnatal growth rates accompanied by decelerated motor skill acquisition and reduced immune function. In the case of mere developmental constraints, we predict that PreGC leads instead to decreased postnatal growth rates in addition to decelerated motor skill acquisition and reduced immune function. PreGC effects on offspring phenotype may be mediated by prenatal food availability, sex of the offspring, maternal caretaking style and postnatal maternal glucocorticoid levels during the lactation period (PostGC) [3,4,8,10,11,20,22,25,48,49]. Hence, if applicable, we controlled PreGC effects on offspring phenotype for these variables as well as potential developmental constraints and trade-offs due to postnatal food availability and investment in (energy demanding physically active) social play [26]. We also consider that increased postnatal growth rates may not be a consequence of accelerated life history but serve to compensate for reduced prenatal growth rate and aim at reducing body size differences at maturation [17,50]. We therefore analyse PreGC effects on offspring body size at age 16–18 months to distinguish between these two possibilities.

## 2. Material and methods

### (a) Study site and subjects

The study was conducted from May 2011 to December 2012 at the Phu Khieo Wildlife Sanctuary in Thailand. Assamese macaques are characterized by female philopatry and male dispersal. Females are fully grown and sexually mature at the age of 5–6 years, and males are fully grown at 9–10 years [26,51]. Average gestation length is 164 days and interbirth interval is bimodally distributed around 14 and 23 months [51]. Female reproduction is seasonal and condition dependent (i.e. probability of conception increases with food availability and female condition [44]). Infant suckling occurs throughout the first 12 months of life (weaning age); however, it occurs at high rates during the first six months only and rates are low during the second six months (electronic supplementary material, figure S1). Therefore, we defined the lactation period here as the first six months of life [44]. We collected data on a fully habituated social group consisting of 24 adults (9 males, 15 females), 4–7 subadult males, 16–19 juveniles (4–8 males, 11–12 females) and 12 infants (6 males, 6 females) born in 2011, and 5 infants (2 males, 3 females) born in 2012. All 17 infants born to 15 mothers were focal animals.

### (b) Data collection

#### (i) Behavioural data

Behavioural data were recorded during 30 min focal animal protocols (1385.4 focal hours, mean ± s.d.: 5.5 ± 0.2 h per individual and month, 86 518 instantaneous records). We recorded instantaneously at 1 min intervals whether the infant was resting, feeding, travelling, socially interacting (affiliative or agonistic) or engaged in solitary or social play. Social play was differentiated from other social behaviours like sitting in body contact, grooming or aggression by the use of a play-face and/or regular role changes. We additionally recorded every minute whether or not the infant was in nipple contact, suckling, or carried by and/or clasped by the mother. We recorded continuously all social interactions of the focal infants. This included approaches and departures into and from 1.5 m proximity, instances of body contact, grooming and agonistic interactions, being restrained from leaving the mother and being refused nipple contact for the first time. In addition, we recorded all aggressive encounters of all group members ad libitum.

#### (ii) Motor skills

For all 17 focal animals, we recorded all occurrences of 18 different motor skills (*n* = 5333 ad libitum records; including closed and open motor skills [52]) to assess individual latencies of skill acquisition (i.e. age at first occurrence for each separate motor skill and individual). All 18 motor skills were acquired by all individuals within the lactation period [26] (electronic supplementary material, table S1).

#### (iii) Growth rate

Size was measured every month via photogrammetry from the length of the lower arm from birth until the end of the study. We took 1706 pictures of the 17 focal animals (6.4 ± 2.1 pictures per individual and month; mean ± s.e.). Picture and object distance were recorded in parallel, and length was calculated by multiplying the object distance with the number of pixels in the picture [26,52] (electronic supplementary material). Outliers (more than mean ± 2 s.d.) were excluded for each month and individual separately, and monthly individual average size and age from the remaining pictures entered the analyses. As linear growth is expected for increase in volume instead of length, we used the cubic value of our length measure (=size index). The relationship between size index and age was linear with normal distribution of residuals, and data were largely unbounded [26] (electronic supplementary material). Growth rates were calculated as slopes of linear regressions of these monthly values over time.

#### (iv) Eye infection

During a two-month outbreak of conjunctivitis during the lactation period, we recorded on a daily basis whether an infant showed external signs of infection or not (electronic supplementary material, figure S3; mean ± s.d.: 22.8 ± 1.6 records per individual). From these data, we calculated the percentage of days an individual had been seen with signs of infection as an approximation of immune function.

#### (v) Availability of ecological energy resources

Monthly food availability indices were calculated based on fruit abundance of 650 trees of the 57 most important food species representing 69% of feeding time for plant matter and the density of these tree species, based on 44 botanical plots within the home range of the study group, covering 20.75 ha of forest. Density was multiplied with phenology scores and summed across tree species to calculate the food abundance index; for details and seasonal variation of food availability in the study site see [44]. Individual energy intake is closely correlated to this index, but not to female rank [44].

### (c) Collection of faecal samples and GC analyses

Faecal samples were collected from mothers during gestation (*n* = 309, mean ± s.d. per female and month: 3.0 ± 1.8; PreGC) and lactation (months 1–6, *n* = 253, 2.5 ± 1.7; PostGC; for details, see electronic supplementary material and [53]). Faecal samples were extracted in ethanol and extracts were analysed for immunoreactive 11β-hydroxyetiocholanolone (GC), a major metabolite of cortisol in primate faeces [55], using enzyme immunoassay. The assay, carried out as described in [56], has been validated for monitoring adrenocortical activity in numerous primate species [55] including Assamese macaques [57,58]. Prior to each assay, extracts were diluted 1 : 200 to 1 : 2000 (depending on concentration) with assay buffer. Assay sensitivity at 90% binding was 2.0 pg. Intra- and inter-assay coefficients of variation, determined by replicate measurements of high- and low-value quality controls, were 5.2% and 9.7% (high), and 7.7% and 13.6% (low), respectively. We ran each sample in duplicate and calculated mass steroid metabolite per mass faecal wet weight in nanograms per gram.

### (d) Statistical analyses

If not stated otherwise, all analyses were run with R v. 3.1.4 [59]. Tests were two-tailed with alpha level set to 0.05. We ensured that test assumptions were fulfilled by computing variance inflation factors (vifs), dffits and dfbetas for all general linear models (LMs), generalized linear models (GLMs) and generalized least-squares models (GLSs; packages car, nlme and piecewiseSEM [60–62]) and visual inspection of scatterplots, residual plots, histograms and Q–Q plots of residuals to check for normality, linearity and homogeneity of variance. All *p*-values were adjusted for multiple testing (function p.adjust with Holm-correction). Only offspring of the first cohort (*n* = 12) was included in the analyses on body size at 16–18 months and conjunctivitis (occurred in first cohort only). All other analyses included all individuals from both cohorts (*n* = 17) with the year of birth as control variable.

Previous studies have shown that PreGC effects on offspring phenotype are often specific for certain gestational trimesters [2,3,7,29,38,39]. Therefore, we analysed PreGC effects on offspring growth not only using the average GC-level throughout gestation, but also using GC levels of each trimester separately (each gestational trimester = 55 days). Based on birth dates (day 0), early gestation ranged from −165 to −111 days, mid from −110 to −56 days, late from −55 to 0 days and early-to-mid from −165 to −56 days.

#### (i) Female rank order

The female dominance rank order was calculated via the I&SI rank order method (Matman 1.1 [63]) on a winner–loser matrix based on dyadic decided conflicts including unprovoked submissions and decided aggressive encounters without mutual aggression or mutual submission [58].

#### (ii) Maternal style

We ran a principal component analysis (SPSS 20.0; IBM) to detect whether and how different types of mother–infant interactions during the lactation period belong to independent maternal style dimensions. We assessed a mother's responsibility for maintaining proximity within 1.5 m to her infant, by calculating the respective Hinde index as the difference between the proportion of approaches by the mother and the proportion of her departures [64], and included this variable into the analysis. As we are not aware of principal component analysis that can implement a control variable, all measures were mean-scaled for year of birth before analysis.

#### (iii) Models 1–6: general description

We ran six different models. Model 1 tested our prediction that PreGC is related to prenatal food availability, and model 2 explored relationships between PreGC and postnatal maternal attributes. Model 3 investigated PreGC-effects on postnatal offspring growth rate and whether these effects are due to PreGC during a certain gestational period. Models 4–6 investigated whether the PreGC effect on offspring growth rate translates into body size differences at 16–18 months and is accompanied by reduced immune function and decelerated motor skill acquisition. Models 3, 4 and 6 included pre- and postnatal food availability, PostGC, maternal caretaking style, sex of the offspring, investment in social play (in proportion of time) and growth rate (only model 6) as control variables. Model 5 had few cases and was only controlled for prenatal food availability. When using repeated measures (models 1, 3, 4, 6) we ran a GLS with ID as grouping variable and (continuous) first-order autoregressive covariance structure (CAR1; model 6: AR1).

If not stated otherwise, postnatal food availability, PostGC and investment in social play were calculated as the average between birth and age at measurement, resulting in different time periods and thus separate values for each data point. For food availability, we first estimated daily indices based on linear interpolation between the monthly indices, and then calculated the average of these daily indices for the respective time period. To control for sampling effort, we included only data points in the analyses that are based on at least three measurements of PostGC on different days and 400 instantaneous records for time spent in social play.

We calculated covariance matrices and vifs. If a predictor of interest had a high vif (greater than 4) and was significant, the null hypothesis had to be rejected, but the estimated effect of the predictor had to be reassessed in a reduced model [65] with the correlated control variable excluded if |*r*| > 0.7.

#### (iv) PreGC and prenatal food availability (model 1)

To analyse the effect of prenatal food availability on PreGC, we ran a GLS with mother ID as grouping variable (*n* = 296 faecal samples) that controlled for maternal rank, offspring sex, year of birth, day of gestation and day time of sampling. We entered a long- and a short-term measure of prenatal food availability in the model (i.e. average food availability) during the three months leading up to the sampling day (‘before’) or on the day prior to faecal sampling (‘present’). Test assumptions were met after log-transformation of the response variable.

#### (v) PreGC and postnatal maternal attributes (model 2)

To analyse correlations between average PreGC during gestation and postnatal maternal caretaking style and physiological stress (PostGC), we ran a LM (*n* = 17) with offspring sex and postnatal food availability (highly correlated to year of birth *r* = 0.994) as control variables.

#### (vi) PreGC-effects on postnatal offspring growth rate (model 3)

We provide a GLS with infant ID as grouping variable (*n* = 227) which quantifies how offspring body size was predicted by an interaction between average PreGC during gestation and age at measurement, controlling for interactions between age and the control variables including year of birth. As many body size measurements were taken after the lactation and PostGC sampling period had ended, we included the average PostGC through lactation instead of time-varying PostGC as control variable.

#### (vii) PreGC-effects on offspring body size at 16–18 months of age (model 4)

We provide a GLS with infant ID as grouping variable (*n* = 34) to investigate the effect of average PreGC during gestation on offspring body size at 16–18 months of age, additionally controlling for age at measurement. The average PostGC through lactation instead of time-varying PostGC was included as control variable.

#### (viii) PreGC-effects on offspring immune function (model 5)

We investigated the effect of average PreGC during gestation on the proportion of days an individual was seen with signs of conjunctivitis during the outbreak applying a binomial logit-link GLM controlling for prenatal food availability (*n* = 12).

#### (ix) PreGC-effects on offspring motor skill acquisition (model 6)

We investigated the effect of average PreGC during gestation on the latencies of skill acquisition with a GLS (*n* = 173) with motor skill labels as categorical control variable and infant-ID as grouping variable. The autoregressive term (AR1) was based on an ordinal motor skill acquisition sequence which was generated by applying the I&SI rank order method (Matman 1.1 [63]) on a before–after matrix (i.e. how often motor skill A was acquired before or after motor skill B over all individuals see [26]). The resulting sequence was highly linear (corrected linearity index *h*′ = 0.998, *p* < 0.001) and the overall consistency of pairwise sequence was 0.716. Five individuals were born more than one week prior to the start of observations and therefore excluded from this analysis.

## 3. Results

### (a) Environmental conditions, maternal characteristics and maternal physiological stress

**Figure 1.**
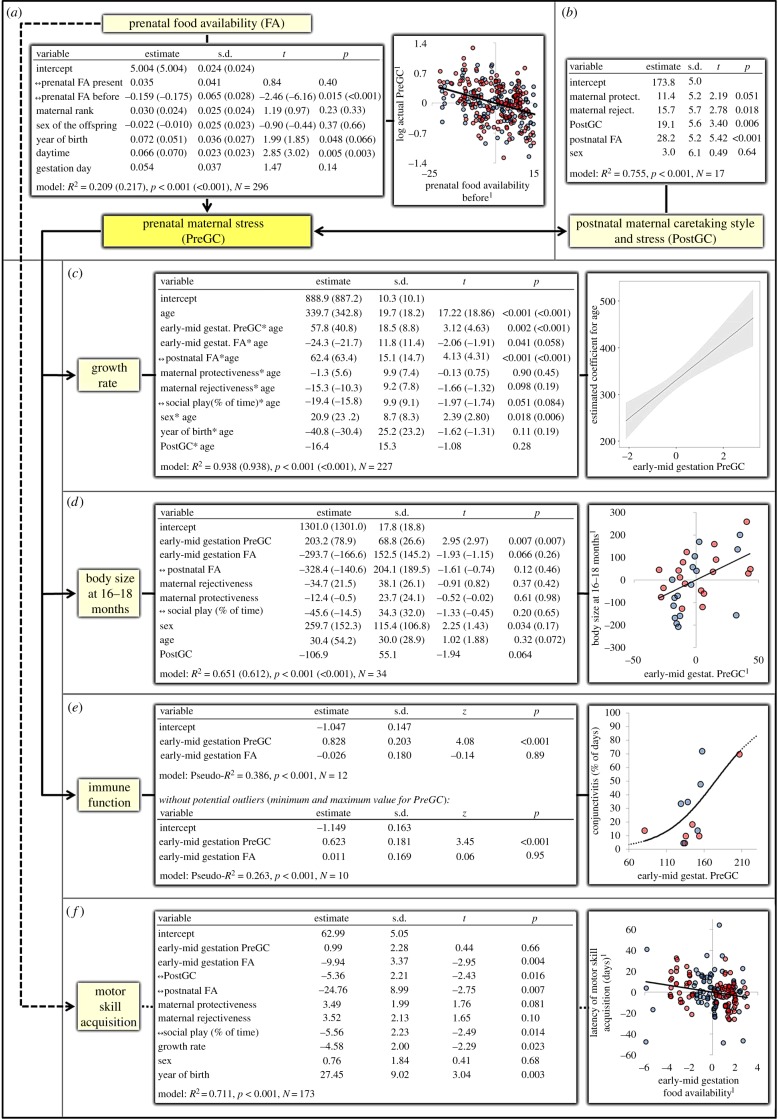
Causes and consequences of maternal physiological stress. Red, females; blue, males. Values in brackets: reduced model after exclusion of the collinear control variable(s) (see text). Superscript 1 in the artwork denotes model residuals (partial regression plot). All fixed effects were *z*-transformed. Sex: male/female = 0/1. (*a*) Prenatal food availability predicted gestational maternal GC level (PreGC) (model 1, GLS, response variable: PreGC (individual samples, log-transformed), grouping variable: mother ID; ↔ on the day the GC in the faecal sample were produced (‘present’) or during the three month leading up to the sampling day (‘before’). (*b*) Postnatal maternal GC level (PostGC) and rejectiveness, and by trend also protectiveness, were independently related to PreGC (model 2, LM, response variable: average PreGC during gestation). (*c*) PreGC during the first and second gestational trimester predicted postnatal growth rates (model 3, GLS, response variable: monthly body size index, grouping variable: infant ID; ↔ from birth until age of separate measurement). We report the main effect for age only because all other main effects do not inform the research question. Chart: the interaction between age and early-to-mid-gestational PreGC of the reduced model is plotted (i.e. the influence of PreGC on the estimate of age; shaded: 95% confidence interval; package: interplot [66]). (*d*) Body size at the age of 16–18 months was predicted by early-to-mid-gestational PreGC (model 4, GLS, response variable: body size indices at 16–18 months of age, grouping variable: infant ID; ↔ from birth until age of separate measurement). (*e*) Proportion of days with signs of conjunctivitis during an outbreak was predicted by early-to-mid-gestational PreGC (model 5, binomial logit-link GLM, response variable: counts of days with signs/without signs). Scatterplot: original data, logit regression line based on model estimates. (*f*) Latency of motor skill acquisition decreased with increasing pre- and postnatal food availability but not early-to-mid-gestational PreGC (model 6, GLS, response variable: individual age at first occurrence, grouping variable: infant ID, with motor skill labels as categorical control variable (not shown); ↔ from birth until age of separate measurement).

First, we analysed potential predictors of current maternal glucocorticoid levels during gestation (PreGC). Controlling for maternal rank, sex of the offspring, year of birth, day of gestation and day time of sampling, we found in the full model that PreGC was negatively correlated to the average longer-term prenatal food availability before sampling but not to the present prenatal food availability at GC sampling (figure 1*a*). Hence, an accumulating effect of food shortages and thus probably reduced maternal physical condition was associated with increased physiological stress during gestation. Prenatal food availability before sampling was highly correlated to present prenatal food availability and day of gestation in the model (*r* = −0.767 and 0.731), which affected its estimate but not its significance (figure 1*a*; vif in full model = 8.91, without present prenatal food availability and day of gestation: vif = 1.65).

Second, we analysed associations between PreGC and postnatal maternal caretaking style and glucocorticoid level during the lactation period (PostGC). The variation in maternal caretaking was characterized by two independent components, which we labelled maternal protectiveness and maternal rejectiveness (table 1). Controlling for postnatal food availability and offspring sex, PreGC was positively correlated to maternal rejectiveness and PostGC, whereas the relationship with maternal protectiveness showed a statistical trend (figure 1*b*).

**Table 1.** Maternal style: principal component analysis. Cut-off value = 0.4, KMO = 0.716, Bartlett's test *p* < 0.001.

principal component analysis	component	
	protectiveness	rejectiveness
close proximity (average duration)	0.908	
body contact (average duration)	0.898	
body contact (total time)	0.841	
clasping (% of time)	0.788	
carrying (% of time)	0.788	
restrain rate	0.640	
Hinde index mother (proximity)	0.554	
aggression rate		0.856
age of refused nipple contact		−0.744

### (b) Maternal physiological stress and offspring attributes

Next, we investigated PreGC effects on postnatal offspring growth rate and whether these effects are due to PreGC during a certain gestational period. PreGC had significant effects on the offspring's growth rate and body size, also after controlling for pre- and postnatal food availability, PostGC, maternal rejectiveness and protectiveness, parallel investment in social play, and sex of the offspring. Offspring growth rate was positively correlated to PreGC during the first and second trimester (early-to-mid-gestational PreGC; figure 1*c*) but not to PreGC during the third trimester (electronic supplementary material, table S2). As a result, early-to-mid-gestational PreGC was a better predictor of offspring growth rate than the average GC levels throughout gestation (electronic supplementary material, table S3). The interactions of age with early-to-mid-gestational PreGC and PostGC were highly correlated in the model (*r* = −0.865), which affected the effect size by but not the significance of the early-to-mid-gestational PreGC effect (figure 1*c*; vif in full model = 9.39, without PostGC: vif = 2.13).

Lastly, we investigated whether this effect on offspring growth translates into body size differences at 16–18 months and is accompanied by reduced immune function and decelerated motor skill acquisition. The positive effect of early-to-mid-gestational PreGC on offspring growth rate led to increased offspring body size at the age of 16–18 months (figure 1*d*), thus constituting a generally accelerated life-history pace instead of a simple catch-up growth. Early-to-mid-gestational PreGC was highly correlated to PostGC in the full model (*r* = −0.930), which strongly affected its estimate but not its significance (figure 1*d*; vif in full model = 14.48, without PostGC: vif = 1.95).

After controlling for prenatal food availability, early-to-mid-gestational PreGC was correlated to the proportion of days with signs of conjunctivitis during an outbreak, which lasted for two months (figure 1*e*).

Latency of offspring motor skill acquisition was not predicted by early-to-mid-gestational PreGC after controlling for pre- and postnatal food availability, PostGC, maternal rejectiveness and protectiveness, parallel investment in social play, sex of the offspring and growth rate (figure 1*f*). Instead, across motor skills the latency of skill acquisition increased with decreasing pre- and postnatal food availability, indicating direct developmental constraints probably due to reduced pre- and postnatal maternal investment.

## 4. Discussion

Our results provide the first evidence for strong effects of elevated prenatal maternal physiological stress (PreGC) on offspring development in a wild non-human primate living under natural conditions, thus adding fundamental data to the sparse literature on PreGC effects in wild animals. We demonstrate that PreGC effects can result from a PAR also in long-lived, slow-developing mammals living in a rather unpredictable environment and being exposed to moderate, evolutionarily relevant stressors only. Although the effects of prenatal maternal stress on offspring adult fitness are beyond the scope of this study, our results provide evidence for the existence of internal, somatic state-based PARs in a species where external, environment forecast-based PARs can be excluded by definition.

The negative relationship between prenatal maternal stress hormone levels and prior food availability in our study suggests that physiological stress was related to maternal physical condition. This probably leads to reduced energy intake of the offspring and developmental constraints, because prenatal maternal stress has been shown to increase with decreasing maternal energy intake and physical condition and to lead to reduced gestational and lactational investment in many mammals [8,20–23]. PreGC effects in terms of developmental constraints due to reduced maternal investment could be reflected in or compensated by postnatal maternal caretaking style [4]. In our study, maternal style during lactation varied along two axes similar to those found in previous studies [67]. PreGC was related to maternal rejectiveness and by trend also to maternal protectiveness which is in agreement with previous findings [8,20,25,49]. Yet the relationships between PreGC and offspring phenotype found in our study were mediated by neither postnatal maternal caretaking style nor glucocorticoid level during the lactation period (PostGC).

PreGC was negatively related to a coarse measure of offspring immune function, supporting previous results [6,7,21,27,28]. We also found decelerating prenatal maternal effects on the offspring's motor skill acquisition, but these effects were due to prenatal food availability instead of PreGC, and probably directly linked to reduced maternal investment. Previous studies reported PreGC effects on motor skill acquisition [6,29] but did not control for maternal energy intake and condition.

The negative prenatal effects on offspring phenotype in our study are in line with predictions from the developmental constraints hypothesis proposing that early adversity constrains offspring development. Developmental constraints lead to disadvantaged adult phenotypes including health deterioration, reduced reproductive success and life expectancy [16,30–35]. The internal PAR hypothesis proposes accelerated growth and reproduction in reaction to developmental constraints and the resulting reduced life expectancy [13,15]. The positive effect of PreGC on offspring growth rate and body size at 16–18 months in our study supports this prediction. Previous results on growth were, however, highly inconsistent, with some studies reporting a positive [14,25,36–38] and others a negative relationship with PreGC [24,25,68] even in the same species. This inconsistency indicates that PreGC is rather invariably related to developmental constraints but does not always induce a PAR. Induction of a PAR might be caused by a stressor's timing during gestation due to varying effects of PreGC on placenta morphology or critical developmental periods, which constrain plasticity [1–3]. The positive effect of PreGC on growth accompanied by the negative effect on immune function in our study suggests that investments into these processes are traded off against each other, and that PreGC rearranges the setting of this trade-off in favour of growth [11,17]. The dataset of this study did not allow us to test this hypothesis directly because data on the age of onset of reproduction, longevity or lifetime reproductive success are not available. Still, previous results suggest that such a trade-off is inevitable because growth and immune function as well as neurodevelopment all strongly rely on available resources [6,17,26,27,69].

Previous research focusing on PARs in long-lived mammals including humans provided no evidence for a PAR and concluded that early adversity may lead solely to developmental constraints and disadvantaged adult phenotypes in such species [30–33,35]. These studies, however, tested predictions for external but not internal PARs [12]. The external PAR hypothesis assumes that individuals with increased adult phenotype–environment matching always outperform mismatched individuals [1,12,13]. Consequently, the above-mentioned studies tested and refuted the resulting critical prediction that under adverse conditions during adulthood, individuals which faced similar (i.e. adverse) early life conditions outperform those which faced different (i.e. optimal) early life conditions. This prediction does not result from the internal PAR hypothesis because early life and adult somatic states are causally linked and adult phenotype mismatches are impossible as long as other confounding variables such as genetic or adult environmental differences are identical [12,13]. Compared with phenotypes of developmentally unconstrained offspring, the phenotype of developmentally constrained offspring will therefore always be disadvantaged. Hence, developmentally constrained adults will hardly outcompete unconstrained ones, but internal PARs will enable them to make the best of a bad job by performing better than without this phenotype recalibration. Results of these previous studies conform to [30–32,35] or even support this view [15,33,34].

Our results, combined with previous results, suggest that PARs in long-lived species are internal rather than external, but external PARs may still apply in short-lived species. Environmental predictability is related to seasonal environmental variation in many of these species and thus potentially high [18]. However, correlations within a stressor over lifetime (e.g. seasonal changes in temperature or food availability) and correlations between stressors (e.g. seasonal population density and individual predation risk) may be positive or negative and can change as season proceeds. Under such complex conditions, it would be expected that external PARs predicting seasonal variation rely on specific prenatal cues rather than the general and unspecific PreGC or environmental adversity. It was shown that offspring maturation in root voles varies with seasonal changes in prenatal maternal melatonin (day length) and chemical by-products of grass ingestion [18]. The strongest evidence for internal and against PreGC-related external PARs comes from studies on cross-generational effects of early life conditions [19]. Effects of PreGC or early life adversity on offspring phenotype in the first generation are passed on to subsequent generations independent from the prenatal environmental conditions of those subsequent generations, in species ranging from short-lived (*Drosophila*, rats) to long-lived (e.g. humans) [1,8,17,19]. While such effects would usually be maladaptive (and easily avoidable) from the external PAR perspective (but see [70]), they conform to internal PARs as mothers facing a disadvantaged adult somatic state due to their own developmental constraints may reduce their maternal investment and thus re-induce developmental constraints in their offspring [19].

## Supplementary Material

Supplementary material

## Data accessibility

Data available from the Dryad Digital Repository: http://dx.doi.org/10.5061/dryad.jq68q [71].
