# Supplementary material for: Prenatal stress effects in a wild, long-lived primate: predictive adaptive responses in an unpredictable environment
Source: Proc Biol Sci. 2016 Sep 28;283(1839):20161304. doi: 10.1098/rspb.2016.1304 (PMC5046897; doi:10.1098/rspb.2016.1304)
Supplement: Supplementary material [file rspb20161304supp1.docx]

**Supplementary material**

**Prenatal stress effects in a wild, long-lived primate: predictive adaptive responses in an unpredictable environment**

**Authors:** Andreas Berghänel, Michael Heistermann, Oliver Schülke, Julia Ostner

correspondence to: [abergha@gwdg.de](mailto:aberghaenel@web.de)

Supplementary methods

Growth data

Picture and object distance were recorded parallel using a Nikon D5000 camera and a Bosch PLR 50 laser distance measurement tool (accuracy ± 2mm). Number of pixels in the picture was determined using ImageJ 1.44p (National Institute of Health, USA). We ran a basic LMM with body size index as response variable, age as predictor variable and immature ID as random effect (N = 227). This model was highly linear (R^2^ = 0.932) and the residuals of this model were normally distributed (Shapiro-Wilcoxon-test: W = 0.991, p = 0.15). The data were largely unbounded at both ends: based on the normal distribution of our data, the probability of a size index ≤ 0 at birth was 0.9% (i.e. the standard distribution of the data were bounded at about -2.4 standard deviations at birth), and the maximal size indices were far from adult body size (figure S2, [26]) with a probability of <0.001% that an immature at the end of the study period has a size index ≥ the minimal adult body size index measured in our study group.

Collection and preparation of faecal samples for GC analyses

Samples uncontaminated with urine or water were collected immediately after defecation, homogenized and approximately 0.5g of faecal material was transferred into a tube containing 4ml of 80% ethanol [53]. Upon return to the field site stress hormone metabolites were extracted from the samples using a validated field extraction protocol following [53]. 2ml of each sample extract was transferred into a polypropylene tube and stored at ambient temperature until transport to the endocrinology laboratory at the German Primate Center Göttingen where samples were stored at -20°C until analysis.

Supplementary figures


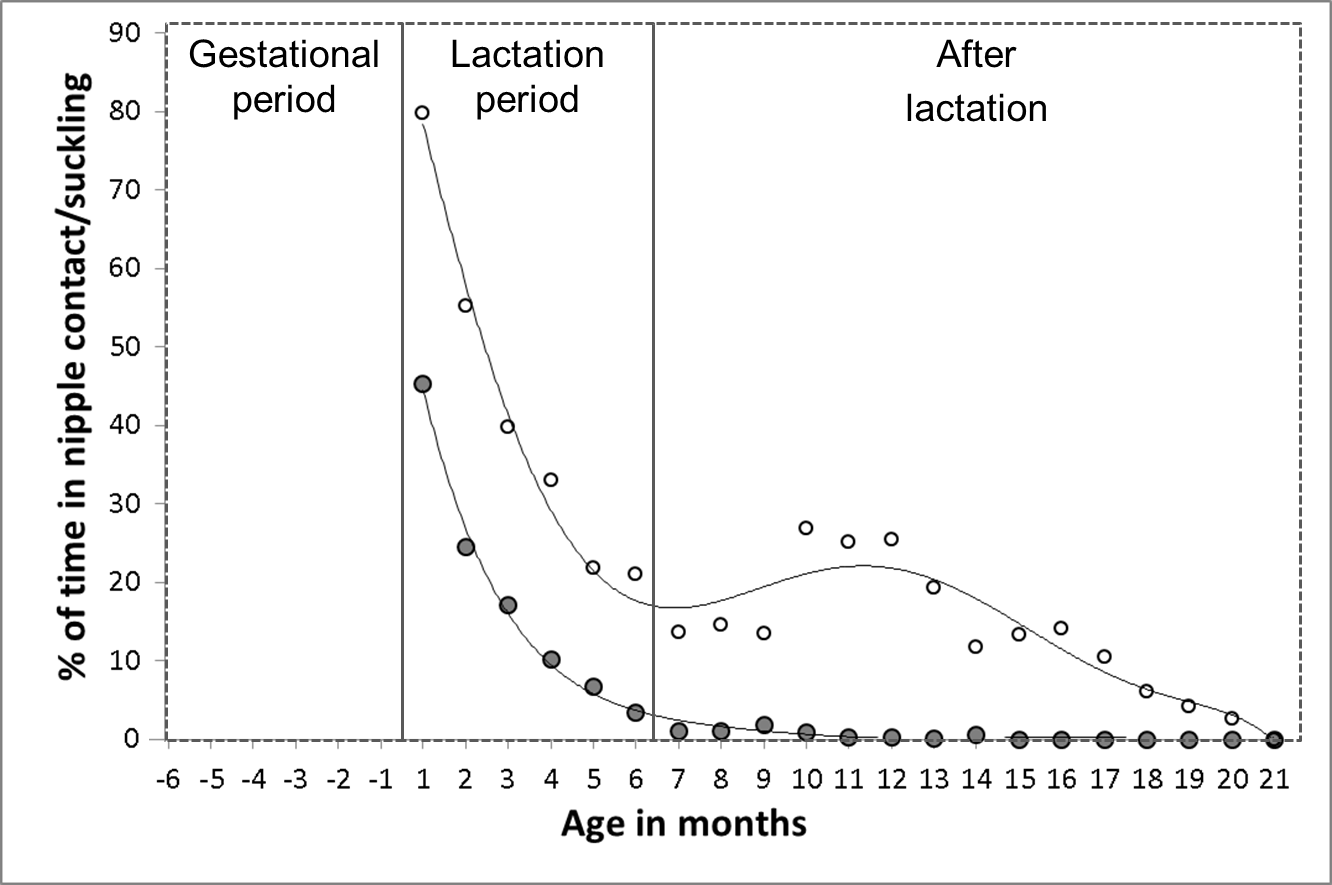


**Figure S1. Frequencies of time in nipple contact (white) and time suckling (grey) over age.**


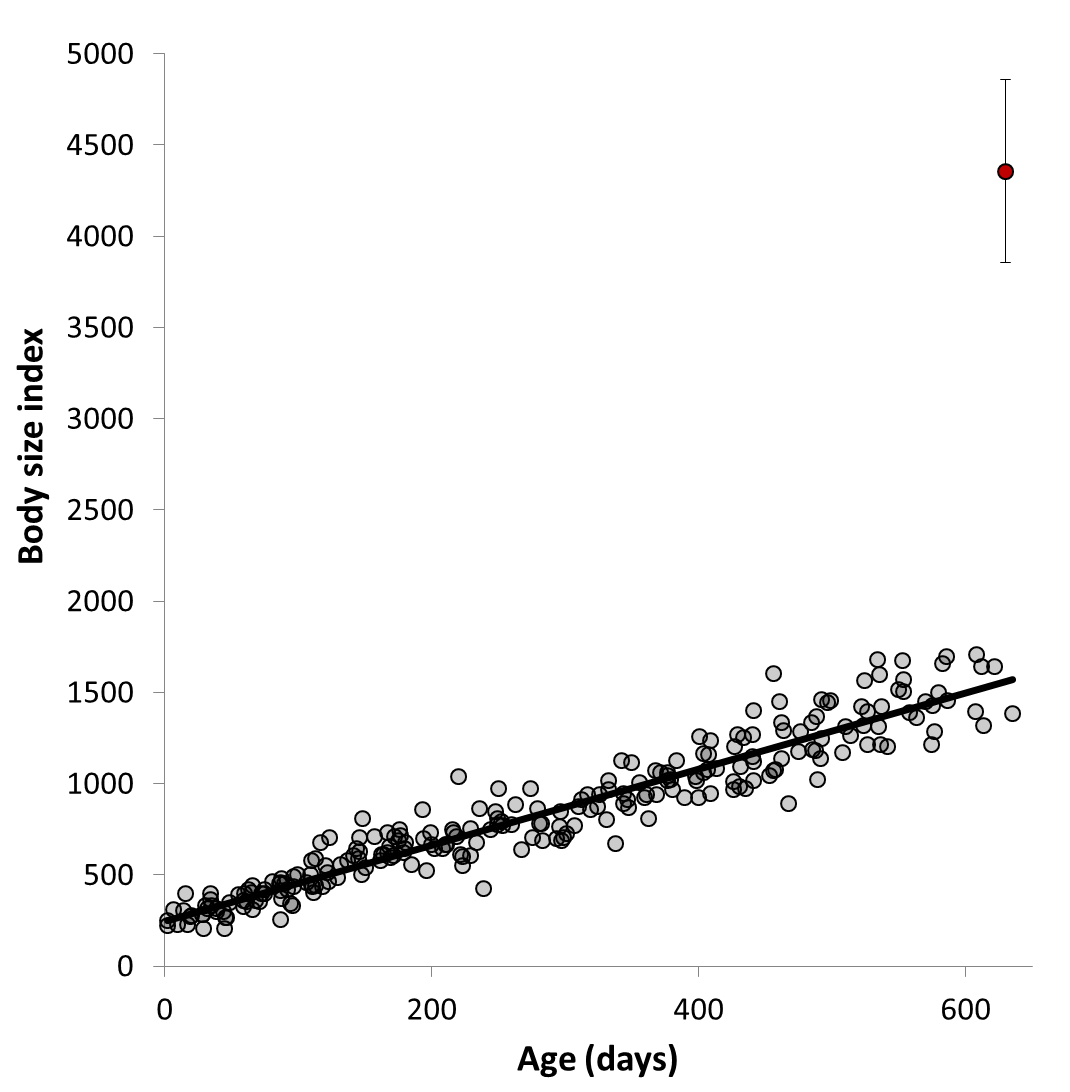


**Figure S2. Body size index increases linearly with age and was far from reaching adult female body size at the end of the study period.** Grey: immatures, red: adult females (mean ± SD).


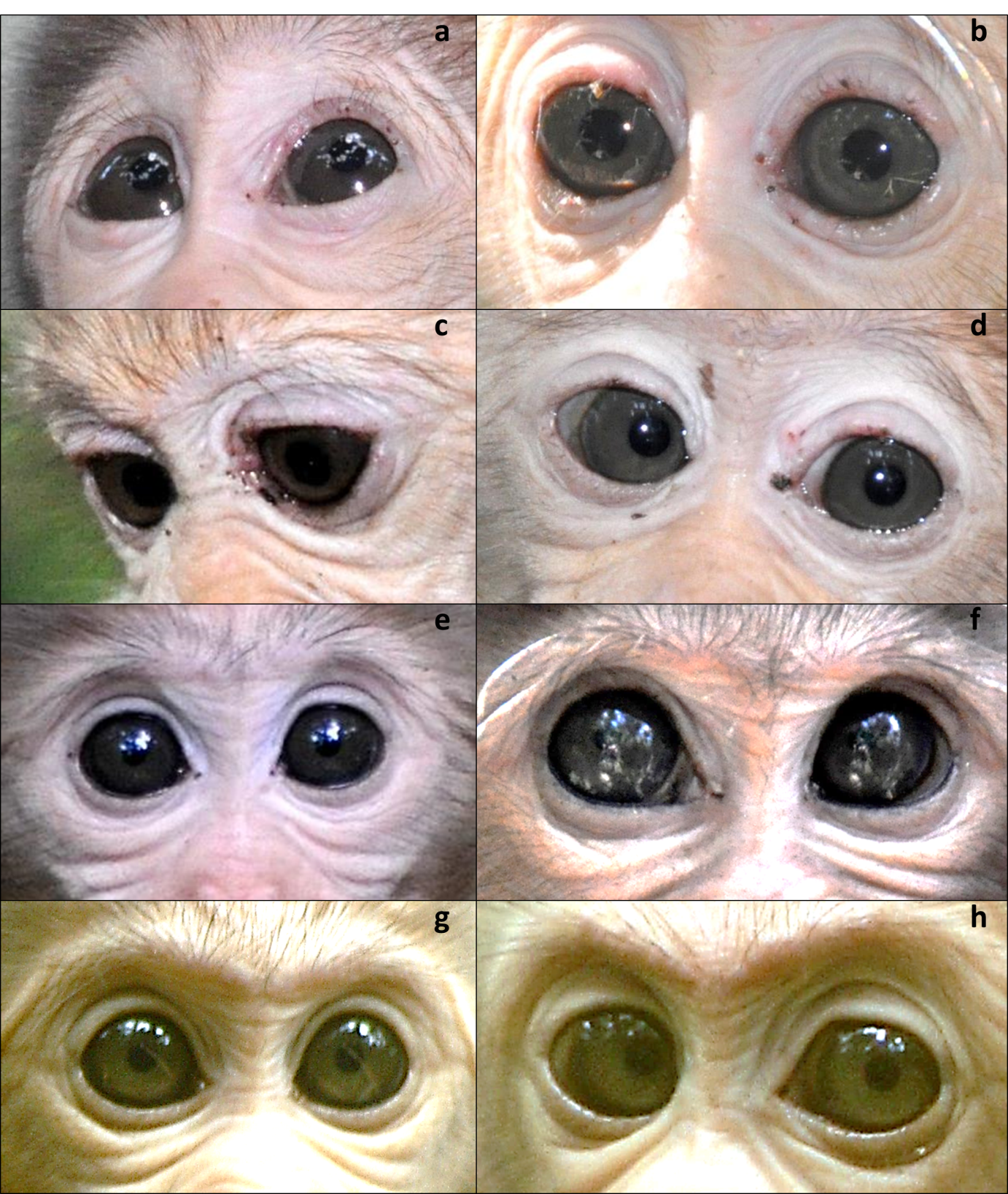


**Figure S3. Conjunctivitis was diagnosed by swelling, reddening and sometimes suppuration of infant conjunctiva.** (a-d) infected infants, (e-h) uninfected infants

Supplementary tables

**Table S1: List of the 18 motor skills used in this study.** * jumping from branch to branch. For more details, see [26].

| Sequence | Motor skill | Average age of acquisition  (Mean±SD) |
| --- | --- | --- |
| 1 | Hanging on all extremities in solitary context | 33.8 ± 15.0 |
| 2 | Hanging on two arms in solitary context | 36.2 ± 14.6 |
| 3 | Jumping on ground | 47.7 ± 15.2 |
| 4 | Hanging on one arm in solitary context | 50.7 ± 20.7 |
| 5 | Jumping in tree | 51.1 ± 15.8 |
| 6 | Running on ground | 54.8 ± 14.5 |
| 7 | Hanging on two legs in solitary context | 59.4 ± 15.3 |
| 8 | Running in tree | 63.9 ± 18.3 |
| 9 | Hanging on all extremities in social play context | 59.8 ± 16.8 |
| 10 | Jumping a distance of <1m in less than 5m height* | 67.6 ± 20.5 |
| 11 | Hanging on two arms in social play context | 62.2 ± 15.7 |
| 12 | Jumping a distance of <1m in more than 5m height* | 67.7 ± 13.3 |
| 13 | Hanging on one leg in solitary context | 71.6 ± 27.3 |
| 14 | Hanging on two legs in social play context | 79.9 ± 19.5 |
| 15 | Hanging on one arm in social play context | 77.8 ± 21.7 |
| 16 | Jumping a distance of 1-2m in less than 5m height* | 105.6 ± 28.7 |
| 17 | Jumping a distance of 1-2m in more than 5m height* | 116.8 ± 27.5 |
| 18 | Hanging on one leg in social play context | 108.4 ± 21.3 |

**Table S2:** **Offspring growth rate was positively correlated to PreGC during the first and second trimester but not to PreGC during the third trimester.** All fixed effects were z-transformed. Sex: male/female = 0/1. Values in brackets: reduced model after exclusion of PostGC. 🡘Time-varying measure from birth until age of separate measurement. Values in brackets: reduced model after exclusion of PostGC*Age

**Table S3:** **Early-to-mid-gestational PreGC was a better predictor of offspring growth rate than the average GC levels throughout gestation.** All fixed effects were z-transformed. Sex: male/female = 0/1. Values in brackets: reduced model after exclusion of PostGC. 🡘Time-varying measure from birth until age of separate measurement. Values in brackets: reduced model after exclusion of PostGC*Age
